# The Anti-SARS-CoV-2 S-Protein IgG, Which Is Detected Using the Chemiluminescence Microparticle Immunoassay (CMIA) in Individuals Having Either a History of COVID-19 Vaccination and/or SARS-CoV-2 Infection, Showed a High-Titer Neutralizing Effect

**DOI:** 10.3390/v16091409

**Published:** 2024-09-03

**Authors:** Dilan Cin, Pinar Soguksu, Meryem Merve Oren, Nuray Ozgulnar, Ali Agacfidan, Sevim Mese

**Affiliations:** 1 Department of Medical Microbiology, Istanbul Medicine Faculty, Istanbul University, 34093 Istanbul, Turkey; dilanncinn@gmail.com (D.C.); soguksup@yahoo.com (P.S.); ali.agacfidan@istanbul.edu.tr (A.A.); 2 Institute of Health Sciences, Istanbul University, 34126 Istanbul, Turkey; 3 Department of Public Health, Istanbul Medicine Faculty, Istanbul University, 34093 Istanbul, Turkey; meryem.oren@istanbul.edu.tr (M.M.O.); nuray.ozgulnar@istanbul.edu.tr (N.O.)

**Keywords:** SARS-CoV-2 IgG, surrogate virus neutralization test (sVNT), chemiluminescent microparticle immunoassay (CMIA)

## Abstract

Neutralizing antibodies plays a primary role in protective immunity by preventing severe acute respiratory syndrome coronavirus-2 (SARS-CoV-2) from entering the cells. Therefore, characterization of antiviral immunity is important for protection against SARS-CoV-2. In this study, the neutralizing effect of the anti-SARS-CoV-2 S1 protein IgG, which was detected using the chemiluminescence microparticle immunoassay (CMIA)-based SARS-CoV-2 IgG II Quant (Abbott, Waukegan, IL, USA) test in SARS-CoV-2 infected and/or vaccinated individuals, was investigated with a surrogate virus neutralization test (sVNT). In total, 120 Seropositive individuals were included in this study. They were divided into two groups: Vaccinated (*n* = 60) and Vaccinated + Previously Infected (*n* = 60). A commercial sVNT, the ACE2–RBD Neutralization Test (Dia.Pro, Milan, Italy), was used to assess the neutralizing effect. The assay is performed in two steps: screening and titration. The screening showed positive results in all seropositive samples. Low titration in 1.7%, medium titration in 5%, and high titration in 93.3% of the Vaccinated group, and medium titration in 1.7% and high titration in 98.3% of the other group, as obtained from the ACE2-RBD titration test. A strong positive and significant correlation was found between the SARS-CoV-2 IgG II Quant test and the ACE2-RBD titration test at the 1/32 titration level for both groups (*p* < 0.001 for both). This study shows that the SARS-CoV-2 IgG detected using the CMIA method after SARS-CoV-2 infection and/or vaccination has a high neutralizing titration by using the sVNT. In line with these data, knowledge that seropositivity determined by CMIA also indicates a strong neutralizing effect contributes to countrywide planning for protecting the population.

## 1. Introduction

SARS-CoV-2, a pandemic agent, develops a humoral immune response within a few weeks after infection, with strong antigenic structural protein properties [1]. The humoral immune response is responsible for the formation of binding antibodies (Babs) and neutralizing antibodies (Nabs) [1,2]. Babs bind to non-virulent epitopes and cannot exhibit the neutralizing effect necessary to defeat the virus [3,4]. On the contrary, antibodies that bind to the surface epitopes of viral particles, which block the entry of the virus into an infected cell and inhibit the virus, are defined as neutralizing antibodies (Nabs) [5,6,7]. While neutralizing antibodies may provide protection from the current disease, they are also associated with long-term protection via the creation of a memory immune response. Therefore, some authors suggest that neutralizing antibody titers can be used as a “correlation of protection” marker [7,8]. The development of Nabs against SARS-CoV-2 following infection or vaccination is important for the development of adequate protection against the coronavirus disease-2019 (COVID-19) [9,10]. To characterize protective antiviral immunity following a SARS-CoV-2 infection or vaccination, a better understanding of the relationship between the binding antibody and the neutralizing effect is required [11].

Early in the pandemic, many commercial kits were developed to measure anti-S-protein receptor binding domain (RBD) antibody concentrations in standard units via calibration using patient serum titers required to reduce the cytopathic effects of the live SARS-CoV-2 Wuhan reference strain in cell cultures [1,12,13]. The immunoassays that are available for detecting exposure to SARS-CoV-2 or vaccine immunization are based on the detection of serum immunoglobulin (Ig) A, IgM, IgG, or total antibodies relative to the virus. These tests, which are based on an enzyme-linked immunoassay, a chemiluminescence immunoassay (CLIA), a chemiluminescent microparticle immunoassay (CMIA), an enzyme-linked fluorescent assay, and a fluorescent microsphere immunoassay, have been granted Emergency Use Authorization (EUA) by the Food and Drug Administration. Although most are qualitative, immunoassays that quantitatively measure SARS-CoV-2 antibodies are also available. Chemiluminescence-based immunoassays have high sensitivity and specificity. The sensitivity and specificity for the CLIA are 77–100% and 90–100%, respectively [14].

The serodiagnosis of SARS-CoV-2 Nabs needs to be explored for an accurate and reliable diagnosis. Viral neutralization tests are important tests used to measure long-term protective immunity after vaccination or natural infection [10]. The conventional neutralization test (cVNT) based on living viral particles is considered a gold-standard reference for the determination of neutralizing activity in patient sera [14]. However, these tests take a long time and are labor-intensive, they can be applied only in biosafety level 3 laboratories, and they are difficult to standardize and implement on a large scale [15]. A pseudovirus is a chimeric virus consisting of a core structure surrounded by the surface protein of the related virus. Internal genes of the pseudovirus are changed to prevent them from synthesizing their own surface proteins, thus discouraging second-cycle replication. For this reason, a pseudovirus-based viral neutralization test (pVNT) can be performed in biosafety level 2 laboratories located in many facilities. However, a pVNT using plasmid and cell culture in the transinfection stages is expensive, and it also requires intensive labor and a long test duration [14,16].

To replace the use of a live virus and cells, which are dependent on expensive and inaccessible BLS3 facilities, diagnostic manufacturers pushed for the launch of commercial binding assays. These assays use sequences of the RBD S protein that are representative of SARS-CoV-2 immobilized on microtiter plates in ELISA or beads in automated CLIA, and this is termed the “surrogate” virus neutralization test (sVNT). The principle of an sVNT is based on the detection of serum and plasma antibodies that block the interaction of the SARS-CoV-2 spike protein RBD with its angiotensin-converting enzyme 2 (ACE2) receptor. This test is performed using an ELISA platform with microplates coated with a recombinant RBD protein. The binding assays were mostly verified and validated via a comparison of ELISA binding to the cell-based neutralizing activity in cell cultures infected with live SARS-CoV-2 using NEQAS or WHO sample sets. The antibody levels in large, randomized cohorts of COVID-19 patients after recovery from SARS-CoV-2 infection, or after two-dose or three-dose vaccinations, were only examined after the regulatory approval of the sVNT ELISA without direct assessments of neutralizing antibody titers per se in cell cultures infected with live variants of concern (VOCs) [15,16,17,18]. For this reason, the reporting of Nabs using an sVNT has often been misleading or incorrectly interpreted. However, recently, the sVNT was adapted to VOCs (B.1, Delta, Omicron BA.1, BA.2 and BA.5) by some researchers and was shown to correlate well with the cVNT [19].

Studies have shown that the sVNT has high sensitivity and specificity. In comparison with a cell-based neutralization test of an sVNT, a high specificity of 99.2% (95%CI: 96.9–99.9) and an overall sensitivity of 80.3% (95%CI: 74.9–84.8) were found for the sVNT [16]. In a comparative analysis of 71 samples with a pVNT to confirm the performance of the sVNT, the agreement between the two methods was found to be 97.2% [20]. In another study that compared three viral neutralization tests, it was shown that the sVNT had 99.93% specificity and 95–100% sensitivity, and the performance of the sVNT correlated well with the performance of both the cVNT and the pVNT [21].

The main purpose of this study is to evaluate the neutralizing effect of SARS-CoV-2 IgG antibodies detected by the CMIA test using an easily applied and low-cost sVNT. It is well known that the serological tests used in routine laboratories detect SARS-CoV-2 IgG antibodies in total without distinguishing between their neutralizing and binding properties. Although some studies have shown a strong correlation between binding antibodies and neutralization, it is still frequently emphasized in the literature that serological tests do not provide information about the neutralizing effect [3,4] However, considering there are a limited number of studies on this subject in Turkey, this study will contribute to the data evaluating the neutralizing effect of SAR-CoV-2 IgG antibodies detected by CMIA in vaccinated and/or infected individuals. At the meantime it will be a model for countries with inadequate economic and physical conditions for using an sVNT, which is a cheap and easy method for this evaluation.

## 2. Materials and Methods

### 2.1. Study Group

The study group consisted of individuals who underwent a SARS-CoV-2 IgG test at the Virology and Basic Immunology Laboratory of Istanbul University, Istanbul Faculty of Medicine, between January 2022 and September 2022. With reference to another study, our study group was filtered as seropositive according to the SARS-CoV-2 IgG test results [22]. In addition, the study group was divided into two subgroups with different causes of seropositivity: Vaccinated and Vaccinated + Previously Infected. A total of 120 individuals, 60 in each group, were included in the study. First of all, individuals who were determined to be SARS-CoV-2 IgG-positive in our laboratory were asked for written consent stating that they volunteered to participate in the study. Individuals who provided this consent filled out a form that questioned their demographic information, whether they had COVID-19, their symptoms, SARS-CoV-2 PCR results, and vaccination status. The PCR results of volunteers with a history of COVID-19 were confirmed using the laboratory’s operating system and in accordance with the consent of these individuals. Individuals who met the inclusion criteria according to the information provided in this form were included in the study.

The inclusion criteria for both groups were determined as follows:

- Over 18 years old and under 65 years old;
- Vaccinated with at least two doses of Sinovac or Biontech vaccines;
- No more than six months have passed since vaccination or infection;
- No chronic or metabolic disease;
- No COVID-19-like symptoms.

For the Vaccinated + Previously Infected Group, in addition to these inclusion criteria, the condition of having an infection within a period of at least four weeks and at most six months was required. The volunteer’s statement was taken into account for past infection. Additionally, with the volunteer’s permission, positive PCR test results were checked using the hospital’s operating system. The last negative PCR test result at an interval of two days was considered as a past infection.

The exclusion criteria for both groups were determined as follows:

- Under 18 years old and over 65 years old;
- Have not been vaccinated with at least two doses of Sinovac or Biontech vaccines;
- More than six months have passed since vaccination or infection;
- Have a chronic or metabolic disease;
- Have COVID-19-like symptoms;
- A PCR test result that has not yet become negative.

**Figure 1:**
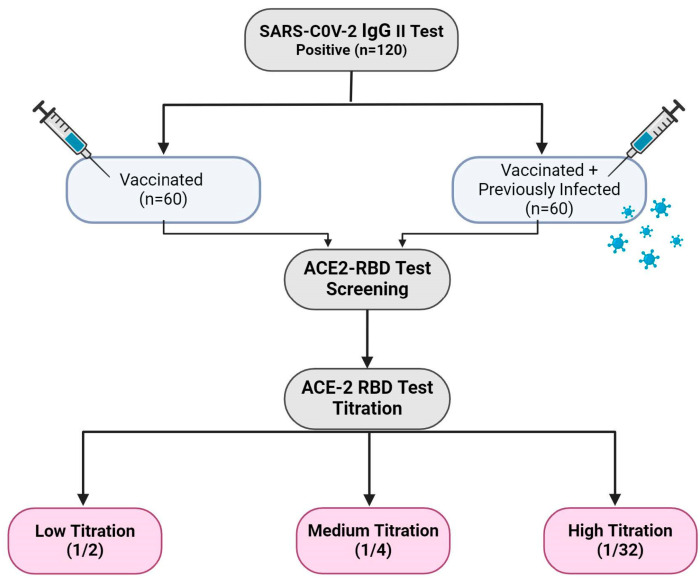
The working plan of the study group. The study included 120 individuals who had SARS-CoV-2 IgG antibodies. Seropositive individuals were divided into two groups: Vaccinated (*n* = 60) and Vaccinated + Previously Infected (*n* = 60). ACE2 RBD screening and titration tests were applied to the SARS-CoV-2 IgG-positive samples (Created with BioRender.com).

Within the scope of the study, the sVNT was applied to investigate the neutralizing effect of SARS-CoV-2 IgG antibodies in the serum samples of individuals determined to be seropositive. The sVNT was applied for screening and titration. After the samples were determined to be positive using the screening test, the samples were diluted, and titration was measured (Figure 1).

Additionally, negative and positive controls provided by the manufacturer were used to ensure the reliability of the tests.

### 2.2. Collection and Storage of Samples

At least 5 mL of blood from individuals determined to be seropositive were collected into gel tubes containing a clot activator, and the samples were kept at room temperature for 30 min to allow clotting. The samples were then placed in a Rotina 38 (Hettich, Kirchlengern, Germany) centrifuge device at 2000× *g* for 10 min to separate the serum. The serum samples were divided into three 1.5 mL Microtubes (SARSTEDT, Nümbrecht, Germany) and stored at −20 °C in a freezer (UGUR, Istanbul, Turkey) to prevent them from being affected by freeze–thaw events.

### 2.3. Chemiluminescence Microparticle Immune Assay (CMIA)

The SARS-CoV-2 IgG II Quant kit (Abbott, Waukegan, IL, USA) was used to detect the presence of specific IgG antibodies that appeared against the S1/RBD of the SARS-CoV-2 S protein in serum samples. Studies were performed with the Architect plus i2000 SR (Abbott, USA) device based on the CMIA method. The steps of the test procedure were performed according to the manufacturer’s instructions. The serum level of SARS-CoV-2 IgG was quantitatively measured in AU/mL with the kit used. According to the kit’s instructions, the results were evaluated as negative if <50 and positive if >50. In the package insert of the kit, the upper limit of quantitation is stated as 40,000 AU/mL. Samples with a SARS-CoV-2 IgG value greater than 40,000 AU/mL were reported as >40,000 AU/mL.

### 2.4. Surrogate Virus Neutralization Test (sVNT)

The sVNT-based ACE2-RBD Neutralizing Assay (Dia.Pro, Italy) was used to determine the neutralizing activity in serum samples with a positive SARS-CoV-2 IgG II test. Within this test, the neutralizing effect of the antibodies is based on the ELISA measurement of the competitive inhibition of binding between the ACE2 receptor and RBD via spike antibodies. The ACE-2 Neutralization Assay (DREF. ACE2-RBD NEUTR.CE 96 Test) contains microplates coated with recombinant glycosylated RBD comprising traditional strain sequences [23]. The serum samples are incubated in the microplates to allow the spike antibodies to bind to the RBD. If the samples do not contain antibodies, the free RBD on the microplates is determined via the addition of ACE2-labeled recombinant biotin followed by Streptavidin–HRP. The color intensity of the bands formed via the binding of ACE2 to RBD is measured using OD450nm. The color intensity is directly proportional to the amount of free RBD in serum samples without spike antibodies [23].

In our study, both screening and titration tests were performed. For each sample dilution, 1/2, 1/4, and 1/32 OD450-620 nm values were measured. The relative binding activity was determined to be positive according to the formula Co/S > 1 or negative according to Co/S < 1, where Co/S is the ratio of OD450–620 nm of the C = cut-off and OD450–620 nm is the ratio of the S = sample. At the dilution (titer) where the Co/S > 1 value is reached, the results are interpreted as follows: low (1/2), medium (1/4), and high (1/32) titer. These titration categories are classed as an sVNT OD, which has been calibrated relative to the titers in cell-based assays by the manufacturer. The sensitivity and specificity of the kit are both given as 100% in the kit’s package insert. The use of NEQAS or WHO sample sets in the performance analysis of sVNT-based tests can be considered as a limitation for sensitivity and specificity due to the risk of excluding VOCs that are in circulation.

### 2.5. Statistical Method

In descriptive statistics, continuous data are provided with the mean standard deviation, median, and minimum and maximum values.

The normality of continuous data was assessed using the Kolmogorov–Smirnov test. A comparison of continuous nonparametric data in independent groups was made using the Mann–Whitney U test in the presence of two groups. The relationship between continuous variables was evaluated using Spearman correlation analysis, where correlation coefficients were considered weak when ranging from 0.0 to 0.24, moderate when ranging from 0.25 to 0.49, strong when ranging from 0.50 to 0.74, and very strong when ranging from 0.75 to 1.00. Categorical data were compared using the chi-square test. For statistical significance, a *p*-value below 0.05 was accepted as significant at the 95% confidence interval. The SPSS v 21.0 program and GraphPad Prism 9.5.1 software were used for statistical analyses.

## 3. Results

### 3.1. Demographic Properties of the Study Groups

The study groups were subdivided according to vaccination and/or infection. The first study group, which included only vaccinated individuals, was defined as “Vaccinated”; and the second group, which included vaccinated and previously infected individuals, was defined as “Vaccinated + Previously Infected”. The median age of the 120 individuals included in the study was 41 (19–63), and the mean was 39 (SD = 13). When the two groups (Vaccinated and Vaccinated + Previously Infected) were evaluated separately, the median age in both groups was 41 (20–63 in the former and 19–60 in the latter). The mean age values of the groups were found to be 41 (SD = 13) and 38 (SD = 11), respectively. There was no statistically significant difference between the two groups with respect to age (*p* = 0.222). While 48.3% of the Vaccinated group consisted of men, with 51.7% being women, the ratio of men and women in the Vaccinated + Previously Infected group was 50%. There was no statistically significant difference between the two groups with respect to sex (*p* = 0.855).

### 3.2. The Results of the SARS-CoV-2 IgG II Quant Test and ACE2-RBD Neutralization Assay

All ACE2-RBD screening test results of a total of 120 samples were found to be positive. For quantitative evaluation, the ACE2-RBD titration test was applied to these samples. According to the results of the ACE2-RBD titration test, the following results in the Vaccinated group were obtained: 1.7% (1/60) low, 5% (3/60) medium, and 93.3% (56/60) high titration. In the Vaccinated + Previously Infected group, the following results were obtained: 1.7% (1/60) medium titration and 98.3% (59/60) high titration (Table 1). While the median value of the SARS-CoV-2 IgG levels of individuals with low and medium titration was determined as 461.2 AU/mL (83.3–1395.2), it is observed that the median value of individuals with high titration was at a very high level of 7352.15 AU/mL (539.7–40,000).

**Figure 2:**
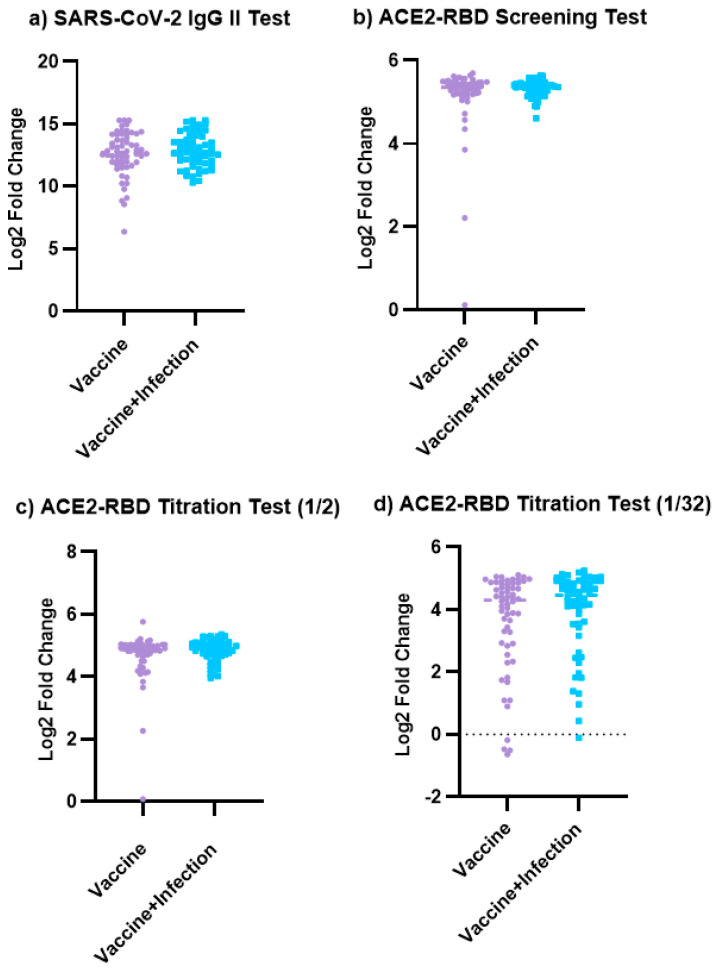
Comparison of the result of the SARS-CoV-2 IgG II test and ACE2-RBD Neutralizing Assay in the Vaccinated and Vaccinated + Previously Infected groups. (**a**) Comparison of the SARS-CoV-2 IgG II test in the Vaccinated and Vaccinated + Previously Infected groups (*p* = 0.2998). (**b**) Comparison of the ACE2-RBD screening test in the Vaccinated and Vaccinated + Previously Infected groups (*p* = 0.1587). (**c**) Comparison of the ACE2-RBD screening test in the Vaccinated and Vaccinated + Previously Infected groups at 1/2 titration (*p* = 0.2570). (**d**) Comparison of the ACE2-RBD screening test in the Vaccinated and Vaccinated + Previously Infected groups at 1/32 titration (*p* = 0.1683). GraphPad Prism 9.5.1 software was used for the production of these figures. If “*p*” is greater than 0.05, there is no significant difference between the two groups.

The results of the SARS-CoV-2 IgG test, ACE2-RBD screening, and 1/4 and 1/32 titration tests in the Vaccinated and Vaccinated + Previously Infected groups were compared. No significant difference was detected between the two groups for all tests (*p* = 0.299, *p* = 0.159, *p* = 0.257, and *p* = 0.168, respectively). These findings are illustrated in Figure 2.

In this study, a strong positive and significant correlation was observed between the SARS-CoV-2 IgG II Quant test and the ACE-2 RBD 1/32 titration levels in both the Vaccinated group and Vaccinated + Previously Infected group (r = 0.622, *p* < 0.001; r = 0.529, *p* < 0.001, respectively) (Table 2).

Furthermore, our study revealed a strong positive and significant correlation between the SARS-CoV-2 IgG levels and the 1/32 titration levels in male and female participants (r = 0.529, *p* < 0.001; r = 0.610, *p* < 0.001, respectively) (Table 3).

When investigating the correlation between SARS-CoV-2 IgG and titration levels based on age groups, a strong positive significant correlation was identified between the SARS-CoV-2 IgG levels and 1/32 titration levels in both the 18–40 and 41–65 age groups (r = 0.542, *p* < 0.001; r = 0.566, *p* < 0.001, respectively). However, a significant weak and negative correlation was observed between the SARS-CoV-2 IgG levels and the ½ titration levels in the 18–40 age group (r = −0.297, *p* = 0.021) (Table 4).

## 4. Discussion

Humoral immunity has an important role in protecting against SARS-CoV-2 infection. Therefore, the tests assessing immune responses allow the determination of the seroprevalence of SARS-CoV-2 infection and vaccination strategies [1,10,24]. Although the serological tests detect the presence of the IgG against SARS-CoV-2 with high sensitivity and specificity, the IgG antibodies do not always indicate neutralizing activity [19]. For this reason, antibodies identified as seropositive with the CMIA SARS-CoV-2 IgG II test (Abbot, USA) were tested in ACE-2 RBD ELISA (Dia.Pro, Italy), which was calibrated relative to the titers of neutralizing antibodies measured in cell-based assays of live SARS-CoV-2 infection. Positive results (100%) with the ACE-2 RBD screening test in all seropositive samples were obtained. To evaluate the neutralizing effect quantitatively, the ACE2 RBD titration test was applied to these samples. According to the results of the ACE2-RBD titration test, the following results in the Vaccinated group were obtained: 1.7% (1/60) low, 5% (3/60) medium, and 93.3% (56/60) high titration. In the Vaccinated + Previously Infected group, the following results were obtained: 1.7% (1/60) medium titration and 98.3% (59/60) high titration (Table 1).

In a study comparing seven chemiluminescent and one fluorescence-based SARS-CoV-2 IgG tests with two commercial sVNTs in confirmed COVID-19 and non-COVID cases, excellent sensitivity (95–100%, ≥15 days after onset of illness) and specificity (95.5–100%) were observed for all tests [25]. The fact that all seropositive results in our study were confirmed via the sVNT (100%) was partially compatible with this study. Since serum samples of healthy individuals from before the pandemic do not exist, it was not possible to evaluate the seronegative results. In this context, the samples with weak antibody responses were evaluated to compensate partly for the lack of seronegative samples. Accordingly, the ACE-2 RBD titration test of four samples in the Vaccinated group with SARS-CoV-2 IgG levels lower than 1000 AU/mL resulted in low or medium neutralization. In the Vaccinated + Previously Infected group, one of the two samples with an antibody level of approximately 1000 AU/mL resulted in low titration. All remaining samples resulted in high titration. In total, low titer neutralization was obtained at a rate of 83.3% (5/6) in samples with weak antibody responses. A study using a chemiluminescence immunoassay examined the distribution and relationship of the S1/S2 IgG assay levels relative to microneutralization assay dilutions [26]. For this purpose, the measurement of the SARS-CoV-2 S1/S2 IgG test was divided into three semi-quantitative groups (<40 AU/mL, 40 to 80 AU/mL, and >80 AU/mL). In these groups, 1/160 titration was determined with the microneutralization test at rates of 39%, 56%, and 87% [23]. These results regarding increased titration rates at high antibody levels support the findings of our study. In this context, it is important to compare the CMIA test with the sVNT test, which has been shown to have high sensitivity and specificity in studies, although it is not a reference method.

In our study, SARS-CoV-2 IgG and ACE2-RBD screening and titration tests were compared in the Vaccinated and Vaccinated + Previously Infected groups and no statis-tically significant difference was found. However, the high titration rate was 93.3% (56/60) in the Vaccinated group, while it was 98.3% (59/60) in the Vaccinated + Previously Infected group (Table 1). Low and medium titration rates in the vaccinated group were found to be 6.7% (4/60) and 1.7%100 (1/60), respectively. While no low titration was detected in the Vaccinated + Previously Infected group, medium titration was detected at a rate of 1.7% (1/60) (Table 1). In a study comparing SARS-CoV-2 S1-IgG, N-IgG, and RBD-IgG tests based on the chemiluminescent method in convalescent, vaccinated, and healthy individuals, a significant difference was found between the groups [27]. However, the Vaccinated + Previously Infected group was not evaluated in this study [27]. In a study evaluating the IgG (S-RBD) anti-body response to an mRNA SARS-CoV-2 vaccination in individuals with and without previous SARS-CoV-2 infections, a significant difference was detected between the two groups after the first dose, while no significant difference was detected after the second dose [28]. Since our study included individuals vaccinated with at least two doses, our results are compatible with this study. Malipiero et al. conducted an anti-RBD IgG immunoassay (sCOVG, Siemens, Erlangen, Germany) and an sVNT (Dia.Pro Diagnostic Bioprobes, Milan, Italy) at different time points up to six months after vaccination in 57 healthcare workers (HCWs) [23]. The same sVNT kit brand as ours was used in this study. It was determined that while high neutralizing bioactivity was maintained in almost all individuals for at least six months after vaccination, the values of the anti-S-RBD IgG showed a significant decrease over time [23]. In our study, measurements at different time points were not carried out; however, since the test covered the six-month period after vaccination and infection, the obtained results were consistent in terms of neutralization.

A strong correlation between the SARS-CoV-2 IgG II Quantitative test and the ACE2-RBD titration test at 1/32 titration for both the Vaccinated and Vaccinated + Previously Infected groups was found (Table 2). Similar to our study, a strong correlation was obtained between the two tests in various studies evaluating the neutralization activity of SARS-CoV-2 IgG detected using ELISA or CLIA methods in convalescent and/or vaccinated groups with the sVNT [29,30,31]. Mouna et al. also showed that both CLIA and sVNT immunoassays were good at identifying cVNT serum dilutions ≥1:16 [29]. The presence of a strong correlation despite high dilution may provide a prediction that the neutralization effect of SARS-CoV-2 IgG detected via CMIA will also be strong. However, the negative correlation at the 1/2 titration level between the SARS-CoV-2 IgG test and the ACE2-RBD test for the 45–65 age group could not be explained. Since the character of the antibody response varies with age, more comprehensive studies that can explain this negative correlation are needed.

In a study conducted in Chile, increased S1-RBD IgG levels in recovered COVID-19 patients were found to be correlated with neutralization activities (*p* < 0.01) and exhibited high variability between individuals [32]. For the analysis of S1-RBD IgG levels according to age, they were found to exhibit high dispersion (*p* = 0.015) with no significant difference between groups, except for the two extreme age groups (18–24 vs. 55 and over). No significant difference was found as a result of the analysis according to sex. However, a trend for higher S1-RBD IgG levels was observed in older men compared to the younger group (*p* = 0.0461) [32]. Unlike this study, which only investigated IgG levels by sex and age, the correlation between the SARS-CoV-2 level and the neutralizing effect by sex and age was also examined in our study, and a strong correlation between the SARS-CoV-2 IgG II Quant test level and ACE2-RBD 1/32 titration in the 18–40 and 41–65 age groups was observed (r = 0.542, *p* < 0.001, r = 0.566, *p* < 0.001, respectively). Similarly, both men and women exhibited a strong correlation at the 1/32 titration level (r = 0.529, *p* < 0.001, r = 0.610, *p* < 0.001, respectively). Despite individuals’ variable characteristics, such as age and gender, the strong correlation between the CMIA test, which is routinely used to determine seropositivity, and the sVNT produces comprehensive data for all groups in predictions for the development of preventive measures and strategies based on seropositivity.

Since the viral spike protein mediates viral entry into target cells by interacting with the ACE2 receptor on host cells, it is the primary target of neutralizing antibodies [33,34]. A particularly high number of mutations in the spike protein enabled viral escape from pre-existing immunity acquired via infection or vaccination with earlier variants [35,36,37]. In a study evaluating the duration of vaccine effectiveness against Omicron variants, it was shown that vaccine effectiveness against symptomatic disease decreased more rapidly for Omicron than pre-Omicron variants. In this meta-regression analysis, it was determined that protection against primary vaccination decreased by almost four to six months, while protection after booster vaccination also decreased rapidly, although to a lesser extent than after primary vaccination [38]. The weakening of protective immunity and the emergence of new variants resulted in an increase in infections and reinfections [39]. Therefore, the need to detect a wide range of neutralizing antibodies that appear during vaccination and infection is emphasized in order to monitor protective immunity. Whole virus neutralization testing (VNT), reported as the reciprocal dilution of serum required to inhibit 50% of infection (NT50), is the gold standard for assessing neutralizing antibody titers in patient serum. Since a VNT requires long-term work with intensive labor in BSL-3 facilities, some researchers have developed a new sVNT adapted to VOCs (B.1, Delta, Omicron BA.1, BA.2, and BA.5) [19]. Silva and colleagues demonstrated the analytical performance of the test they developed by comparing it with the VNT kit. Researchers obtained moderate to strong correlations for Omicron sub-variants (Spearman’s r = 0.7081 for BA.1, r = 0.7205 for BA.2, and r = 0.6042 for BA.5), WT (r = 0.8458), and Delta (r = 0.8158) [19]. In this study, researchers emphasized that the sVNT allowed for the effective prediction of immune protection against various VOCs [19]. Due to the budget limitations of our study, the analysis of the variants has not been included. However, in our study it was determined that the dominant variant circulating in Turkey between January and September 2022 was Omicron using GISAID’s data [40]. 

Because of the budget restrictions and physical conditions, one of the limitations of our study was that it was carried out using only an sVNT but not a cVNT or a pVNT. However, this limitation is acceptable since the sVNT exhibited high sensitivity/specificity (≥98% and 100%, respectively) and good correlations (R^2^ ≥ 0.84) in a study in which it was compared with a cVNT and a pVNT in different populations [21]. An additional study was carried out by Lokida et al., in which they evaluated the performance of ten commercial immunoassays with an sVNT [25]. In their study, excellent sensitivity (95–100%, 15 days after the onset of the disease) and specificity (95.5–100%) were obtained for ten tests, and a moderate to high correlation was observed with a GenScript sVNT (r = 0.58 to r = 0.98), except for one. The researchers suggested that commercial serological tests are useful for assessing immune responses after infection and vaccination; however, they argue that their reliability should be evaluated regularly [25]. In countries where economic and physical conditions are not sufficient, an sVNT is advantageous as an alternative to a cVNT and a pVNT, which require technical infrastructures and high costs. Accumulation of data showing that SARS-CoV-2 IgG antibodies detected by routine serological tests have a strong neutralizing effect allows seropositivity to be evaluated more confidently in terms of protection. Thus, verification based on data that seropositive individuals in the community also have neutralizing antibodies contributes to the development of nationwide protective measures and strategic plans.

## 5. Conclusions

In conclusion, seropositivity determined with the SARS-CoV-2 IgG II test was confirmed with an ACE2-RBD Neutralizing Assay, which is an sVNT. A strong correlation was detected between the two tests at the 1/32 titration level for both the Vaccinated and Vaccinated + Previously Infected groups. A titration test is recommended in addition to the ACE2-RBD screening test to evaluate the neutralization of SARS-CoV-2 IgG positivity detected at low levels.

The conclusion of our study contributes to the existing data against the statements that serological tests do not provide information about neutralization. Considering the limited number of studies on the subject in Turkey, our data are even more important. Thus, these data can inform estimates of protection and vaccination strategies based on the seropositivity determined using routine binding antibody tests.

## Figures and Tables

**Table 1:** Distribution of the results of ACE2-RBD titration and SARS-CoV-2 IgG II tests in the Vaccinated and Vaccinated + Previously Infected groups.

ACE-RBD Titration Test	Vaccinated Group (*n* = 60)		Vaccinated + Previously Infected Group (*n* = 60)	
	SARS-CoV-2 IgG II Test		SARS-CoV-2 IgG II Test	
	50–5000 AU/mL	>5000 AU/mL	50–5000 AU/mL	>5000 AU/mL
Low	1 (1.7%)	0 (0%)	0 (0%)	0 (0%)
Medium	3 (5%)	0 (0%)	1 (1.7%)	0 (0%)
High	0 (0%)	56 (93.3%)	0 (0%)	59 (98.3%)

SARS-CoV-2: Severe acute respiratory syndrome coronavirus-2. IgG: Immunoglobulin G. ACE2-RBD: Angiotensin-converting enzyme 2–receptor binding domain.

**Table 2:** Correlation between the SARS-CoV-2 IgG II test and the ACE2-RBD Neutralization Assay levels in the Vaccinated and Vaccinated + Previously Infected groups.

ACE-2 RBD Neutralization Assay						
			Screening	1/2 Titration	1/4 Titration	1/32 Titration
Vaccinated	SARS-CoV-2 IgG II	r	0.083	−0.068	−0.036	0.622
		*p*	0.529	0.604	0.786	<0.001
Vaccinated + Previously Infected	SARS-CoV-2 IgG II	r	−0.131	−0.223	−0.105	0.529
		*p*	0.319	0.087	0.423	<0.001

SARS-CoV-2: Severe acute respiratory syndrome coronavirus-2. IgG: Immunoglobulin G. ACE2-RBD: Angiotensin-converting enzyme 2–receptor binding domain.

**Table 3:** Correlation between the SARS-CoV-2 IgG test and ACE2-RBD Neutralization Assay levels by sex.

Correlation						
			ACE2-RBD Screening	1/2 Titration	1/4 Titration	1/32 Titration
Male	SARS-CoV-2 IgG II	r	−0.092	−0.101	−0.028	0.529
		*p*	0.489	0.447	0.833	<0.001
Female	SARS-CoV-2 IgG II	r	0.007	−0.215	−0.117	0.610
		*p*	0.957	0.097	0.370	<0.001

SARS-CoV-2: Severe acute respiratory syndrome coronavirus-2. IgG: Immunoglobulin G. ACE2-RBD: Angiotensin-converting enzyme 2–receptor binding domain.

**Table 4:** Correlation between the SARS-CoV-2 IgG test and the ACE2-RBD Neutralization Assay levels by age.

Correlation						
			ACE2-RBD Screening	1/2 Titration	1/4 Titration	1/32 Titration
18–40 Age	SARS-CoV-2 IgG II	r	0.030	−0.297	−0.056	0.542
		*p*	0.819	0.021	0.669	<0.001
41–65 Age	SARS-CoV-2 IgG II	r	−0.120	−0.028	−0.073	0.566
		*p*	0.363	0.835	0.578	<0.001

SARS-CoV-2: Severe acute respiratory syndrome coronavirus-2. IgG: Immunoglobulin G. ACE2-RBD: Angiotensin-converting enzyme 2–receptor binding domain.

## Data Availability

The data can be shared by the authors, if desired, in accordance with patient confidentiality principles.
